# Microwave Synthesis, Basic Spectral and Biological Evaluation of Some Copper (II) Mesoporphyrinic Complexes

**DOI:** 10.3390/molecules15053731

**Published:** 2010-05-25

**Authors:** Rica Boscencu, Mihaela Ilie, Radu Socoteanu, Anabela Sousa Oliveira, Carolina Constantin, Monica Neagu, Gina Manda, Luis Filipe Vieira Ferreira

**Affiliations:** 1 Faculty of Pharmacy, “Carol Davila” University of Medicine and Pharmacy, 6 Traian Vuia St., 020956 Bucharest, Romania; 2 “Ilie Murgulescu” Institute of Physical Chemistry, Romanian Academy, 202 Splaiul Independenţei, 060021 Bucharest, Romania; E-Mail: psradu@yahoo.com (R.S.); 3 Centro de Química-Física Molecular, Institute of Nanosciences and Nanotechnology, Instituto Superior Técnico Av. Rovisco Pais 1049-001, Lisbon, Portugal; E-Mails: asoliveira@estgp.pt (A.S.O.); luisfilipevf@ist.utl.pt (L.F.V.F); 4 Escola Superior de Tecnologia e Gestão de Portalegre, Instituto Politécnico de Portalegre, Apartado 148, 7300-901 Portalegre, Portugal; 5 “Victor Babeş” National Institute for Pathology and Biomedical Sciences, Bucharest, 99-101 Splaiul Independenţei, 050096 Bucharest, Romania; E-Mail: caroconstantin@yahoo.com (C.C.)

**Keywords:** copper (II) mesoporphyrinic complexes, FT-IR spectroscopy, solvatochromy, cytotoxicity

## Abstract

Cu(II) complexes with asymmetrical and symmetrical porphyrinic ligands were synthesized with superior yields using microwave irradiation. The paper presents the synthesis of 5-(3-hydroxyphenyl)-10,15,20-tris-(4-carboxymethylphenyl)-21,23-Cu(II)-porphine in comparison to its symmetrical complex 5,10,15,20-*meso*-tetrakis-(4-carboxy-methylphenyl)-21,23-Cu(II) porphine. The two compounds were characterized by FT-IR, UV–Vis and EPR spectroscopy, which fully confirmed the structures. The spectral molecular absorption properties of the porphyrinic complexes were studied in organic solvents (methanol, ethanol, *iso*-propanol, dimethyl sulfoxide, dimethylformamide and methylene chloride), and the influence of the solvent polarity on the absorbance maxima is described. In order to establish their future potential in biomedical applications preliminary toxicological studies consisting of viability and proliferation of standard tumor cell lines (MCF7 and B16) testing was performed. The obtained results indicate a low toxicity for both compounds and further recommends them for testing in light activation protocols.

## 1. Introduction

Porphyrins and metalloporphyrins represent one of the most widely studied of all known macrocyclic ring systems [1]. The synthesis of metalloporphyrins and the investigation of their chemical and biological properties has attracted increased interest in both bioinorganic chemistry and medical chemistry, mainly due to their possible use in unconventional treatment of various diseases by means of photodynamic therapy (PDT) [2], which is a selective method of treatment of several diseases, amongst which the most cited are cancer and psoriasis. The technique involves the administration of a pharmaceutical formulation containing a specific photosensitizer, its selective localization at the level of the sick cells, laser irradiation of the photosensitizer-loaded tissue, followed by the generation of a very reactive chemical species, the singlet oxygen (^1^O_2_), that destroys the cells [3,4,5,6,7,8,9]. The most important advantage of photodynamic therapy consists in the use of visible light range, having much lower energies compared to those used in the classical radiological therapy.

The first porphyrins used in the PDT were the *hematoporphyrin derivatives* (HPD). A purified HPD fraction (the sodium porphymer) represents the active component of the pharmaceutical formulation PHOTOFRIN^®^, used in the treatment of esophageal cancer. The results obtained using PHOTOFRIN^®^ in PDT proved that it presented a series of disadvantages as follows: its composition included several inactive PDT derivatives; it was slowly eliminated from healthy tissues so patients remained photo-sensitive to the solar radiation, in certain cases more than a month after the procedure. However, the greatest disadvantage of PHOTOFRIN^®^ is connected to its spectral properties, its strongest light absorption band being situated in a rather inefficient spectral range for PDT [2,10,11,12]. 

A second generation of photosensitizers includes porphyrinic structures as tetra-(*m*-hydroxyphenyl) chlorine or *m*-THPC*,* the active substance of the pharmaceutical formulation FOSCAN^®^, used in the treatment of head and neck cancers. The doses for an individual administration are rather low compared to PHOTOFRIN^®^ (0.1 mg/kg body weight) and the irradiation wavelength is at λ = 652 nm [13].

Recently, research has been directed towards obtaining new photosensitizers that meet the following requirements: a high purity and simple production under laboratory conditions; photoactivity at wavelengths higher than 630–680 nm; acceptable solubility in biologic fluids for an easy localization at the cellular and subcellular level; great selectivity for the malignant or other targeted tissue; lack of toxicity in the absence of the exciting light; rapid elimination from the body after the treatment is performed and non-toxic metabolites [14,15,16,17,18,19,20,21,22].

**Figure 1:**
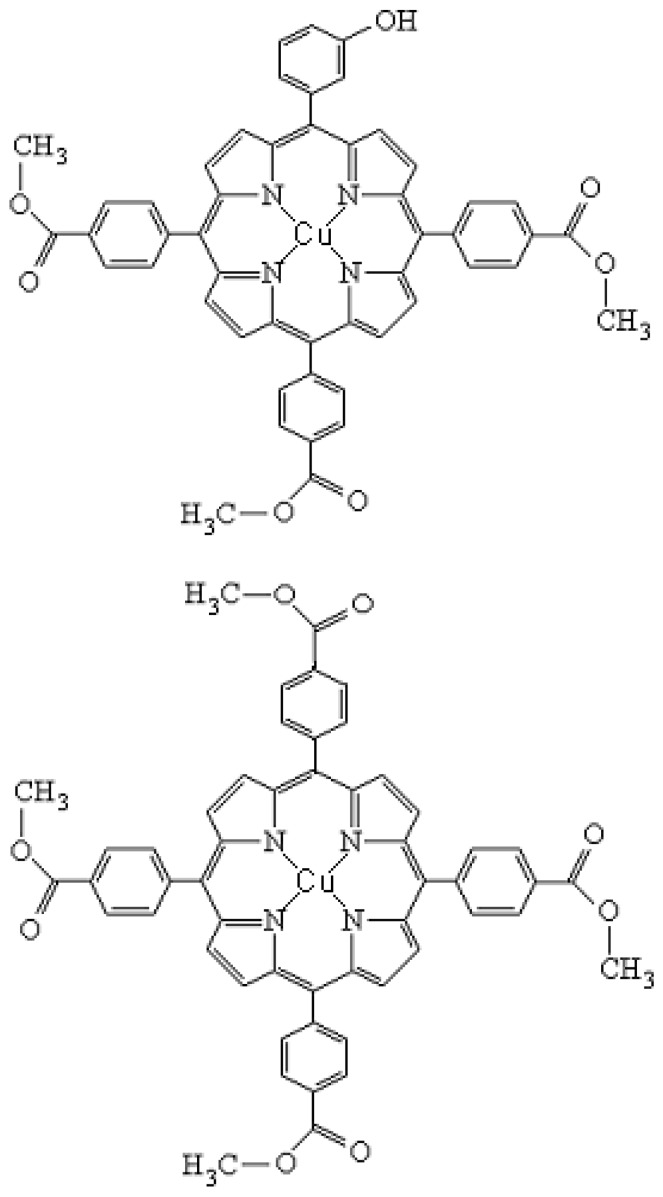
Structures of 5-(3-hydroxyphenyl)-10,15,20–*tris*-(4-carboxymethylphenyl)–21, 23 Cu(II) porphine (Cu(II)TCMOH_m_) (top) and 5,10,15,20-*meso*-tetrakis-(4-carboxy-methylphenyl)-21,23-Cu(II) porphine (Cu(II)TCMP) (bottom).

This paper presents the characteristics of a newly synthesized asymmetrical Cu(II) porphyrinic complex, namely 5-(3-hydroxyphenyl)-10,15,20-*tris*-(4-carboxymethylphenyl)-21,23 Cu(II) porphine (denoted as Cu(II)TCMPOH_m_ - Figure 1) and a comparison to the corresponding symmetrical compound - 5,10,15,20-*meso*-tetrakis*-*(4-carboxymethylphenyl)-21,23-Cu(II) porphine (denoted as Cu(II)TCMP - Figure 1), obtained using microwave irradiation, aiming at further investigation of the theoretical and preclinical aspects of their possible use in PDT.

## 2. Results and Discussion

### 2.1. Infrared spectra

The important band frequencies of the ligand and copper complexes and corresponding assignments are presented in Table 1. The IR spectra of the porphyrinic ligands TCMPOH_m_ and TCMP were analyzed in a previous paper [23]. The IR bands of the ligands at approx. 3,310 cm^-1^ and 963 cm^-1^ are due to the N-H stretching and bending vibration of the porphyrinic core, but they disappear in the complexes because the hydrogen atom in the N-H bonding is replaced by the Cu(II) ion. The bands at 3,503 cm^-1^ are assigned to O-H stretching vibration of the –OH functional group in the TCMPOH_m_ and Cu(II)TCMPOH_m_ . Other bands observed in the higher wavenumber region (2,957–2,923 cm^-1^) are due to the stretching vibration of C-H bond of the porphyrinic ring. The bands of the compounds in the range of 1,715–1,720 cm^-1^ are assigned to C=O stretching vibrations. The IR spectrum of the free-base porphyrins and Cu(II) complexes clearly indicates the presence of the -O-CH_3_ group in the spectral range 2,848–2,853 cm^-1^.

**Table 1:** Characteristic IR vibrations of the free base porphyrins and their Cu(II) complexes (KBr pellet).

Characteristic vibration	Wavenumber of the IR band (cm^-1^)			
	TCMP^1^	Cu(II)TCMP	TCMPOH_m_^1^	Cu(II) TCMPOH_m_
ν_O-H_	-	-	3503 *m*	3493 *m*
ν_N-H_	3310 *w*	-	3312 *m*	-
ν_C-H_	2950 *m*	2954 *m*	2952 *m*	2957 *m*
ν_C-H_	2923 *m*	2924 *m*	2923 *v.s*	2923 *m*
ν_C-H_ from -O-CH_3_	2851 *s*	2853 *s*	2853 *m*	2848 *s*
ν_C=O_	1717 *v.s.*	1715 *s*	1718 *s*	1720 *m*
ν_C-N_	1603 *m*	1604 *m*	1597 *s*	1600 *s*
ν_C-H_ pyrrole	1400 *m*	1402 *w*	1401 *m*	1410 *w*
ν_C-O_	1156 *s*	1164 *m*	1156 *s*	1164 *m*
δ_C-H_	1018 *m*	1014 *m*	1020 *w*	1010 *w*
δ_N-H_ pyrrole	963 *m*	-	964 *m*	-
γ_C-C_		869 *w*	858 *w*	862 *w*
γ_C-N_ pyrrole	798 *m*	797 *s*	790 *m*	795 *s*

The intensities of the signals are described as weak *(w),* medium *(m),* strong *(s)* and very strong *(v.s.)*; ^1^ Data for this compound were taken from [23].

### 2.2. Molecular electronic spectra

**Figure 2:**
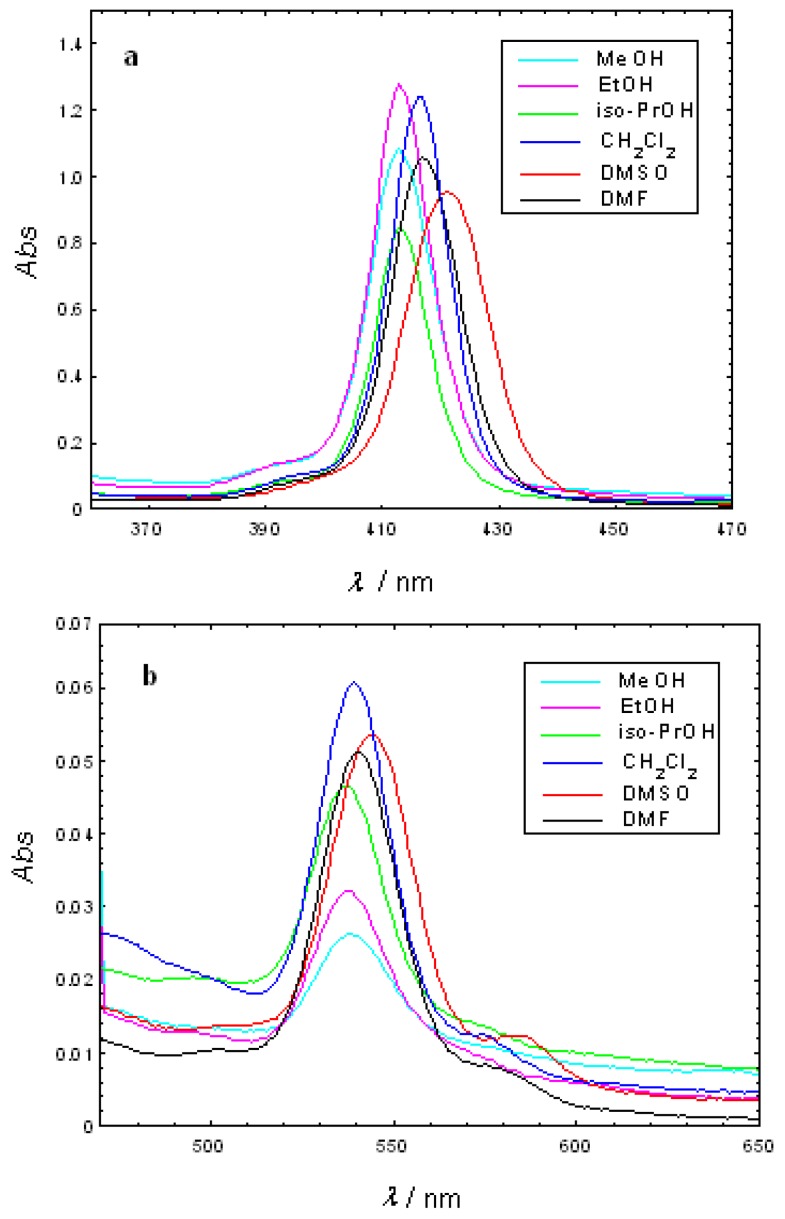
Absorption spectra of 2.5 × 10^-6^ M Cu(II)TCMP in different solvents (**a**-Soret band; **b**-Q bands).

**Figure 3:**
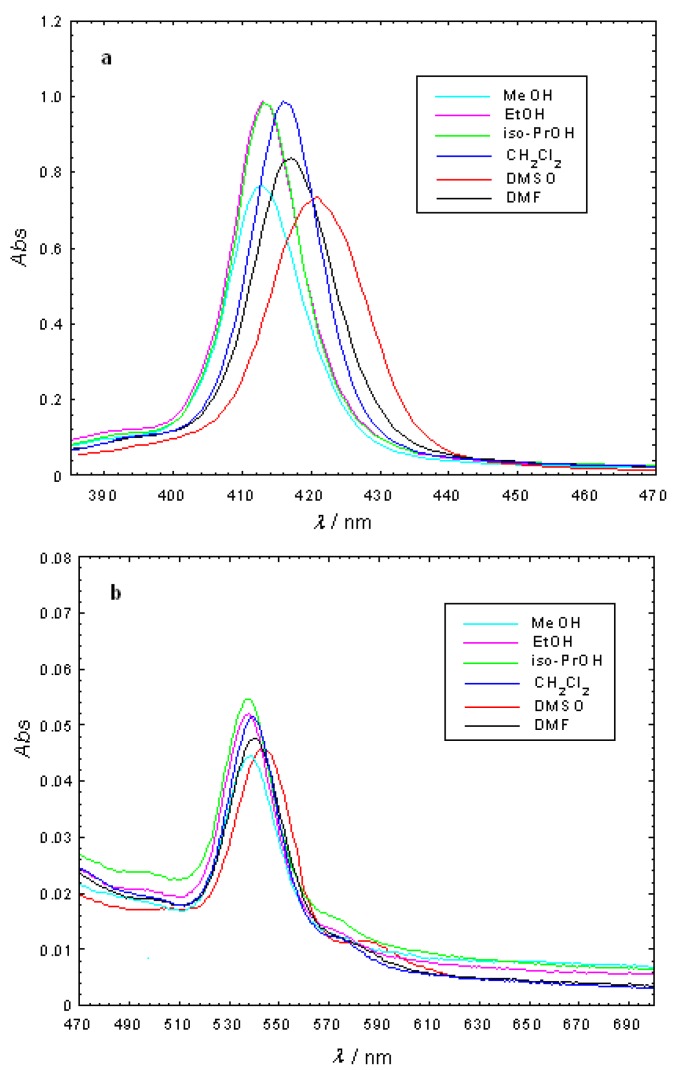
Absorption spectra of 2.5 × 10^-6^ M Cu(II)TCMPOH_m_ in different solvents (**a**-Soret band; **b**-Q bands).

Molecular electronic spectra were studied for the free-base porphyrins, Cu(II)TCMP and Cu(II)TCMPOH_m_ in different solvents (methanol - MeOH, ethanol - EtOH, isopropanol - *iso*-PrOH, dimethylsulfoxide - DMSO, dimethylformamide - DMF, dichloromethane - CH_2_Cl_2_). For this purpose, porphyrin solutions were freshly prepared in spectrally pure solvents at a concentration of 2.5 × 10^-6^ M. Solutions were kept in dark to prevent photodegradation. All the absorption spectra were recorded for the same samples. The obtained results are presented in Figure 2 and Figure 3 and Table 2.

**Table 2:** Maximum wavelength and molar absorptivity of the porphyrinic ligands, Cu(II)TCMP and Cu(II)TCMPOH_m_ in different solvents (c = 2.5 × 10^-6 ^M).

Solvent	λmax (nm) [lgε (L mol^-1^ cm^-1^)]				
	Soret B(0,0)	Q_y_(1,0)	Q_y_(0,0)	Q_x_(1,0)	Q_x_(0,0)
**5, 10, 15, 20-*meso*-tetrakis-(4-carboxymethylphenyl) - 21,23-H porphine**					
MeOH	415.3 [5.528]	514.8 [4.415]	544.3[4.387]	591.9[4.265]	646.9 [4.342]
EtOH	416.4 [5.546]	512.5 [4.428]	547.4 [4.283]	590.4 [4.225]	647.4 [4.146]
*iso*-PrOH	416.8 [5.511]	513.5 [4.301]	546.9 [4.049]	592.4 [3.857]	648.4 [3.602]
CH_2_Cl_2_	419.6 [5.699]	515.2 [4.344]	550.0 [4.000]	590.1 [3.806]	645.5 [3.643]
DMSO	420.9 [5.602]	515.6 [4.310]	550.0 [4.049]	590.1 [4.000]	645.6 [3.833]
DMF	419.3 [5.662]	514.3 [4.326]	548.7 [4.017]	589.6 [3.833]	645.3 [3.716]
**5, 10 ,15 ,20–*meso*-tetrakis-(4-carboxymethylphenyl)-21,23-Cu(II)porphine**					
MeOH	412.5 [5.637]	-	538.2 [4.024]	-	-
EtOH	413.0 [5.709]	-	537.7 [4.107]	-	-
*iso*-PrOH	413.1 [5.527]	-	537.0 [4.274]	574.8(sh)	-
CH_2_Cl_2_	416.4 [5.697]	-	539.4 [4.387]	574.5(sh)	-
DMSO	421.6 [5.582]	-	544.1 [4.334]	584.6 [3.681]	-
DMF	417.0 [5.627]	-	540.3 [4.310]	578.0 (sh)	-
**5- (3-hydroxyphenyl)-10, 15, 20–tris-(4-carboxymethylphenyl) – 21,23-H porphine**					
MeOH	422.4 [5.582]	513.1 [4.158]	554.8 [4.193]	595.5 [3.924]	647.6 [3.643]
EtOH	424.1 [5.561]	513.4 [4.134]	556.3 [4.158]	596.1 [3.857]	649.4 [3.556]
*iso*-PrOH	424.6 [5.577]	513.1 [4.146]	556.0 [4.182]	596.7 [3.881	650.3 [3.602]
CH_2_Cl_2_	420.1 [5.607]	515.2 [4.121]	548.8 [4.146]	588.9 [3.716]	647.9 [3.556]
DMSO	429.3 [5.546]	516.1 [4.170]	558.6 [4.182]	598.8 [3.944]	648.8 [3.681]
DMF	426.5 [5.519]	514.9 [4.107]	556.8 [4.121]	597.9 [3.833]	648.2 [3,556]
**5-(3-hydroxyphenyl)-10, 15, 20-tris-(4-carboxymethylphenyl)-21,23- Cu(II)porphine**					
MeOH	412.7 [5.486]	-	538.3 [4.255]	-	-
EtOH	413.1 [5.598]	-	537.1 [4.318]	-	-
*iso*-PrOH	413.6 [5.593]	-	537.7 [4.342]	570.3(sh)	-
CH_2_Cl_2_	416.1 [5.598]	-	539.5 [4.310]	-	-
DMSO	421.0 [5.468]	-	544.0 [4.265]	585.9(sh)	-
DMF	417.0 [5.525]	-	540.0 [4.283]	579.6(sh)	-

sh – shoulder; MeOH= methanol, EtOH=ethanol, *iso*-PrOH=isopropyl alcohol, DMSO=dimethylsulfoxide, DMF=dimethylformamide, CH_2_Cl_2 _= dichloromethane.

The electronic absorption spectrum of the free-base porphyrins is dominated by a typical Soret band and four Q bands located in the spectral range 415–650 nm, depending on the solvent [23]. The Q bands of the free base porphyrins consist of four absorption peaks which are typical to the Qx(0,0), Qx(0,1), Qy(0,0), Qy(0,1) transitions in the free base porphyrin (D_2h_ symmetry). After the metal ion entered into the body of the porphyrin, the number of Q bands decreases and the absorption frequencies shifts due to the increasing of the molecular symmetry from D_2h_ to D_4h_. 

The UV-VIS spectra of the Cu(II)TCMPOH_m_ and Cu(II)TCMP exhibited one Soret band in the spectral range of 412–421 nm accompanied by two Q bands, respectively in the 538–544 nm and 570–585 nm spectral range. Also, in the Cu(II)TCMP and Cu(II)TCMPOH_m_ the Q bands are hypsochromically shifted and partially overlapped, as already reported in the literature for other metalloporphyrins of the same type [14,24,25,26,27]. 

It should be noticed that the wavelength of the Soret band maximum changes in the various solvents. As well, the relative intensities of the Soret band to the Q bands differ in some solvents. The Soret band of TCMP is shifted by about 3 nm, whereas this shift is larger for TCMPOH_m_, Cu(II)TCMPOH_m_ and Cu(II)TCMP (about 9 nm). That could be explained by the stronger interactions of the porphyrinic compounds with the solvent dipoles. The changes in Soret and Q bands can be associated with the solvent polarity [28] and the interactions between the porphyrinic substitutents and the solvent molecule.

The blue-shift of the bands with increasing polarity of the solvents should be noticed for porphyrinic ligands and their Cu(II) complexes. Thus, the absorption bands of the ligands and Cu(II) complexes in alcohol was blue-shifted which can be ascribed to the formation of proton bridges between the alcohol molecules and the porphyrinic compounds due to the polarity-induced and proton properties of the alcohol.

### 2.3. EPR spectra

EPR spectra of the newly synthesized complexes were recorded on powders at room temperature. It provides information about the environment of the Cu(II) within the porphyrinic ligand and the coordination geometry. The EPR spectrum of the synthesized complexes indicates the presence of the Cu(II) in tetrahedral surroundings with D_4h_ symmetry.

The values of the magnetic parameters *g_||_*, *g_⊥_*, *A_||_* and *A_⊥_* for Cu(II)TCMPOH_m_ and Cu(II)TCMP were calculated and are presented in Table 3. These magnetic parameter values, obtained from the EPR spectra of Cu(II) with the porphyrinic ligands are close to those reported in the literature [29,30]. Also, the *g_||_* value (*g*_|| _< 2.3) indicates a covalent character of the Cu-N bonds in the copper-porphyrinic complexes [31].

**Table 3:** Magnetic parameter values corresponding to Cu(II) complex combinations with porphyrinic ligands.

Complex combination	*g_||_*	*g* *_⊥_*	*A_|| _* x *10^4 ^* (cm^-1^)	*A* *_⊥ _* x *10^4 ^* (cm^-1^)
Cu(II)TPPOH_m_^1^	2.113	2.055	202	28
Cu(II)TCMP	2.162	2.042	203	32
Cu(II)TCMPOH_m_	2.170	2.045	201	33

^1^ Data for this compound were taken from [25].

### 2.4. Preliminary toxicological tests

**Figure 4:**
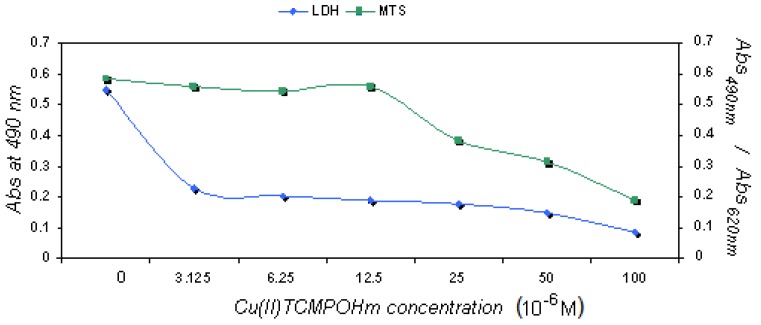
Cellular response of MCF7 line after 24 h incubation with Cu(II)TCMPOH_m._

As the foreseen application of the compounds will be PDT, we have used standard cell lines that originated from tumors like skin melanoma and breast cancer that can be treated by photodynamic therapy. Different incubation times were used in order to test the short and longer term effects on the cell physiology. For MCF7 line, after a short 2 h incubation no major effect was registered in the concentration ranges, but after 24 h at higher doses (> 50 μM), the LDH release was slightly enhanced while proliferation was reduced (Figure 4), mainly for Cu(II)TCMPOH_m_.

**Figure 5:**
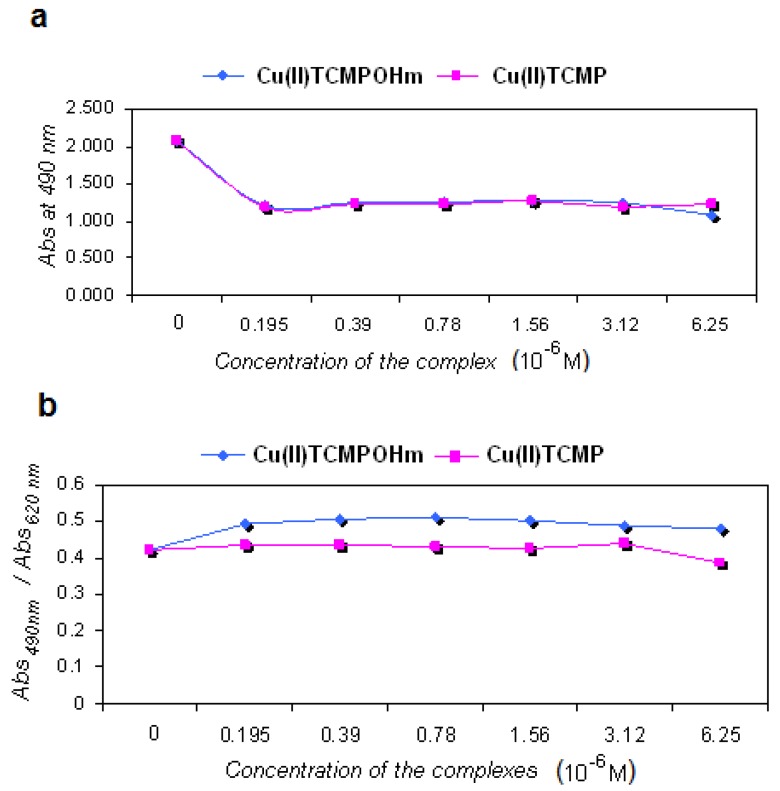
Cellular response of B16 line after 2 h incubation with Cu(II)TCMPOH_m_ and Cu(II)TCMP (**a **– viability, **b** – proliferation).

However at low doses of both copper complexes, the viability and proliferation were not related to the applied dose, results that support the low toxicity of both compounds. Regarding B16 skin melanoma standard cell line, the results were quite similar to those obtained for MCF7 line (Figure 5), namely after short periods of incubation no major effect is registered. After 24 h of incubation in low doses this cell line is as well not affected by the compounds. The lack of dark toxicity on a large concentration domain recommends these compounds for further testing in experimental approaches for activation upon light irradiation. 

## 3. Experimental

### 3.1. Materials and Methods

Commercially available chemicals and solvents were used as received from Sigma-Aldrich and Merck. IR spectra were recorded with a FT-IR 400D Nicolet Impact spectrophotometer. The substances under analysis, previously dried for 24 h at 150 ºC, were processed as KBr (spectrally pure) pellets. The spectra were recorded in the 4,000–500 cm^-1^ spectral range.

The molecular absorption spectra were recorded on a Lambda 35 Perkin-Elmer spectrophotometer in solution, using a 10 mm path length quartz cell (Hellma), in single beam mode. UV-Visible spectra were obtained for Cu(II)TCMP and Cu(II)TCMPOH_m_ in different solvents: MeOH, EtOH, *iso*-PrOH, DMSO, DMF, CH_2_Cl_2. _The porphyrin solutions were freshly prepared in the spectrally pure solvents at the concentration 2.5 × 10^-6^ M. The solutions were kept in dark to prevent photodegradation.

EPR spectra of the newly synthesized complexes were recorded using a ART-6 spectrometer, operating in the X band (9.01 GHz), equipped with a field modulation unit of 100 KHz. Mn(II) salt in MgO was used as a reference for the magnetic field calibration. The spectra were recorded on powders at room temperature.

For preliminary toxicological studies viability and proliferation in presence of copper porphyrinic complexes was performed on standard cell lines provided by the European Collection of Cell Cultures (ECACC) as following: MCF7 (human mammary adenocarcinoma cell line – ECACC 86012803) and B16 (murine cutaneous melanoma cell line – ECACC 94042254) cells. The compounds were used in sterile conditions after resuspension in DMSO followed by sonication at 22000 Hz for 30 seconds. The stock solutions were diluted in the appropriate culture medium for each cell line at the stated concentrations. The complexes were studied in two concentration ranges (3.125–100 μM and 0.195–6.26 μM), following 2 h. and 24 h incubation with the mentioned cell lines. For viability we have used lactate dehydrogenase (LDH) release test [32], with CytoTox 96^®^ Non-Radioactive Cytotoxicity Test (Promega) and the cell proliferation by means of the tetrazolium salt (MTS) reduction test [33], using CellTiter 96^®^ AQ_eous_ One Solution Cell Proliferation Assay kit (from Promega). Results were expressed as triplicates mean of optical density (OD) at 490 nm for the mentioned viability and proliferation tests.

### 3.2. Synthesis of copper porphyrinic complexes

We have previously reported the synthesis of porphyrinic complexes from the corresponding tetrapyrrolic ligand and appropriate metal salts in the presence of a basic catalyst, 2,6-dimethylpyridine [24,25]. However, in recent years, synthesis via microwave irradiation has attracted considerable attention, as microwave-assisted reactions are believed to facilitate the polarization of the substrates thereby promoting the reactions [34,35,36,37]. Microwave-assisted reactions have become increasingly important in the synthesis of tetrapyrrolic compounds due to advantages such as significant reduction in reaction times and side reactions, increased yields, ease of purification and minimization of the amount of solvent used [38,39]. These are the reasons why in this paper the synthesis of the copper porphyrinic complexes was carried out using microwave irradiation assisted synthesis.

Methyl 4-formyl benzoate (0.45 mol), 3-hydroxybenzaldehyde (0.15 mol), fresh distilled pyrrole (0.60 mol), copper chloride anhydrous (0.15 mol), 2,6-dimethylpyridine (1 mL) and silicagel 60 (200–500 μm, 35–70 mesh, 8 g) dry silica were mixed at room temperature in a Pyrex bottle. 2,6-Dimethylpyridine is a Lewis base and captures the hydrogen of the porphyrinic ring to produce the porphyrin dianion (P^2^^−^), the copper ion (Cu^2+^) is attracted by the P^2^^−^ to form the copper porphyrin. The mixture reaction was irradiated in a 475W domestic microwave oven at 180 ºC (final temperature), for 8 min. Extraction of samples for monitoring the synthesis was performed after every 2 min of irradiation. The presence of the complex in the reaction mixture was monitored by UV–Vis spectroscopy. The crude product was then purified on chromatography column by several elutions, using dichloromethane as eluent and silica gel (100–200 mesh size) as stationary phase; to obtain the final copper porphyrinic complexes, preparative TLC (on 2 mm silicagel 60 plates) was used. The same procedure was adopted in the preparation of 5,10,15,20-*meso*-tetrakis-(4-carboxymethylphenyl)-21, 23 Cu(II) porphine. The yields obtained were 68% for Cu(II)TCMPOH_m_ and 83% for Cu(II)TCMP, respectively. Following the preparation the copper porphyrins were characterized by IR, UV-VIS and EPR spectrometry. 

To compare the two synthesis procedures, the synthesis of the copper porphyrin complexes was also carried following the method described in a previous paper [33]. Solutions (~10^–4^ M) of the porphyrinic ligands TCMP and TCMPOH_m_ [33] in dichloromethane were gently heated while stirred, until the ligand crystals were completely dissolved. Then, several drops of 2,6-dimethylpyridine were added, together with the appropriate amount of anhydrous CuCl_2 _in methanol solution, to yield the copper metal complexes in a 1:1 molar ratio. The reaction mixture was refluxed, under continuous stirring, for 1 h, at 55 ºC. The presence of the complex in the reaction mixture was monitored during the reaction by thin layer chromatography. After cooling, the reaction mixture was purified passing it through a silica gel chromatographic column. The solutions of the complexes in dichloromethane were concentrated by simple distillation. The violet crystals were dried at about 100 ºC, for 12 h.

The physical-chemical data of copper porphyrins prepared via the two different methods described above are identical; in addition the reactions presented in this paper have been successfully repeated several times with the same results.

## 4. Conclusions

The paper presents the non-conventional synthesis of a new asymmetrical Cu(II) porphyrinic complex, 5-(3-hydroxyphenyl)-10,15,20-tris-(4-carboxymethylphenyl)-21,23 Cu(II) porphine and its corresponding symmetrical compound 5,10,15,20-*meso*-tetrakis(4-carboxymethylphenyl)-21,23 Cu(II) porphine, by means of microwave irradiation assisted solvent-free synthesis. The copper porphyrinic complexes were synthesized to be used as PDT sensitizers.

The spectral properties of the Cu(II) porphyrinic complexes were investigated by FTIR, EPR and UV-Vis spectroscopy. The influence of the solvent polarity on the molecular absorption band maxima was also described. A blue shift of the spectral bands was observed with increasing solvent polarity, determined by interactions between porphyrinic substituents and the solvent molecule.

Preliminary viability and proliferation *in vitro* tests were performed on MCF7 (human mammary carcinoma) and B16 (murine cutaneous melanoma) cell lines, for different doses and incubation times. The results of the biological *in vitro* tests indicated a low cytotoxicity of the compounds for the studied cells. The concentration ranges tested would give valuable information for further settling of a concentration for the compounds to be tested in view of PDT applications.
